# Knowledge & attitudes toward fertility preservation (Medical and social freezing) among Lebanese women between the ages of 18 and 39 years

**DOI:** 10.1371/journal.pone.0291249

**Published:** 2023-09-08

**Authors:** Ghina Ghazeeri, Christine Beyrouthy, Lina El-taha, May Abiad, Duaa Fahs

**Affiliations:** 1 Department of Obstetrics and Gynecology, Faculty of Medicine, American University of Beirut Medical Center, Beirut, Lebanon; 2 Division of Reproductive Endocrinology and Infertility, American University of Beirut Medical Center, Beirut, Lebanon; University Hospital of Münster, GERMANY

## Abstract

Egg freezing is a relatively new and controversial procedure in the Arab region, challenging traditional perceptions of fertility and motherhood. This study aims to assess Lebanese women’s awareness and acceptance of egg freezing and how these attitudes differ according to vary with age, socio-demographic characteristics, and educational level. We conducted a cross-sectional survey targeting Lebanese females aged between 18 and 39, involving 402 Lebanese women from six different institutions representing diverse cultural backgrounds. 65% of the respondents had heard of egg freezing. Younger women (18–30 years old) were 2.09 times more likely to consider egg freezing than those aged 31–39. Single women were 4.31 times more likely to consider egg freezing than women in relationships, while childless women were 5.00 times more likely compared to women who already had children. Overall, medical egg freezing was more widely accepted than social egg freezing. The most supported indication for social egg freezing was to enable women who struggled to find the right partner during their peak fertile years to have children in the future (41.5%). The most common concern that affected women’s decision to undergo egg freezing was whether the procedure would be proven safe for their future children and whether it would affect their future fertility. Interestingly, in a relatively conservative country, concerns about hymenal disruption were the least prevalent, (19%). The most common concern by far was limited information on the procedure (62%). In conclusion, the study reveals that awareness and acceptance of social egg freezing among Lebanese women were higher than expected. Limited information on the procedure’s details was the main impediment to higher acceptance rates, highlighting the importance of physicians and primary healthcare providers in providing reproductive-aged women with the necessary information to safeguard their reproductive potential.

## Introduction

Egg freezing, also known as oocyte cryopreservation, involves collecting eggs, cryopreserving them using vitrification, and storing them for future fertilization by in vitro fertilization (IVF). Initially, egg freezing was only used for women undergoing treatments that negatively affected their ability to conceive, such as gonadal-toxic chemotherapy, radiation, or ovarian surgery. However, the increasing trend of delaying childbearing for financial, personal, psychological, or career-related reasons has paved the way for a new indication for freezing social egg freezing. Population-based birth statistics show a consistent increase in the number of women choosing to postpone childbearing, primarily due to the increasing involvement of women in the labor market at the expense of having children [1]. While there is no single definition of advanced reproductive age in women, numerous population studies have confirmed that the decline in birth rates begins at age 35 [2]. The decline in primordial follicles at reproductive age remains steady at around 1,000 per month until age 37, after which it accelerates [3]. In addition to oocyte quantity, declining oocyte quality plays a pivotal role in the reproductive potential of women older than 35 years due to increasing oocyte aneuploidy rates with age. The rate is low in women younger than 35 (10%) but escalates to 30% at 40, to 40% at 43, and around 100% in women over age 45 [4].

Fertility preservation offers several advantages, such as securing reproductive potential, reducing the number of inefficient treatments undergone at an advanced age, and minimizing the risk of having children with chromosomal abnormalities associated with ovarian aneuploidy. However, recent research has highlighted the impact of other factors on reproductive outcomes, such as female obesity. In a study conducted by Papler et al. in 2019, obesity had a negative effect on the quality of day 5 embryos compared to normal-weight women, despite there being no difference between the two groups in oocyte quality immediately after retrieval [5].

Egg freezing is an ethically acceptable alternative to embryo freezing for women with moral concerns about a developing embryo status [6]. However, like any medical procedure, egg freezing carries some risks, such as ovarian hyperstimulation syndrome, risks of prospective IVF (multiple pregnancies, gestational hypertension, premature delivery, and infants with low birth weight), and risks of pregnancy at an advanced age (premature delivery, operative delivery, multiple pregnancies, low birth weight, gestational diabetes, and hypertensive disorders of pregnancy) [6]. Additionally, there is very limited and conflicting evidence regarding the association between ovarian stimulation and increased risk of ovarian and breast cancer, with the certainty of the evidence, being very low when assessed using the GRADE approach [6, 7].

In 2013, the American Society for Reproductive Medicine (ASRM), published a practice guideline approving the use of egg freezing solely for medical purposes, but cautioned against social freezing ‘‘for the sole purpose of circumventing reproductive aging in healthy women” due to limited data on its “safety, efficacy, emotional risks, and cost-effectiveness” [8]. However, with emerging reassuring evidence on the efficacy of egg freezing, the ASRM has since published a new Ethics Committee Opinion stating that “Planned oocyte cryopreservation is an ethically permissible medical treatment that may enhance women’s reproductive autonomy and promote social equality. However, uncertainties exist regarding any new treatment’s efficacy and long-term effects. Patients considering this treatment must be apprised of these unknowns” [9].

Numerous studies have been conducted to assess attitudes towards egg freezing across different countries. A German study including 643 female and male participants showed that the majority of participants had a negative attitude toward social freezing (52%), while 34% were positive and the rest had a neutral attitude (14%) [10]. Another survey conducted in Belgium that included 1049 women between the ages of 21 and 40 showed that 31.5% would consider social freezing, 51.8% would not consider it, and 16.7% had a neutral attitude [11]. In both studies, almost half of the participants have negative attitudes compared to one-third of positive attitudes. This limited overall acceptance of the procedure is further portrayed in studies assessing the percentage of women considering themselves to be potential social oocyte freezers: 27%, 21.6%, and 19.5% in the UK/Denmark [12], US [13], and Italian [14] cohorts respectively.

To date, there are no studies conducted in the Arab region regarding this topic. Therefore, we aim to explore knowledge and attitudes toward fertility preservation in the context of social norms among a specific population encompassing a wide diversity of backgrounds.

## Materials and methods

This is a cross-sectional study that included Lebanese females between 18 and 39 years old. A questionnaire was sent to students and employees at seven different institutions across different Lebanese areas from January 2019 to June 2019. The questionnaire was prepared based on formerly validated questionnaires published in journals that reflect attitudes associated with fertility preservation while contemplating cultural and religious concerns. Surveys were completely anonymous, and participants were notified about their right to withdraw at any point without any further implications. After explaining the study, participants were asked for their written consent.

An online survey was sent to participants from the American University of Beirut (AUB), the AUB Medical Center, the Beirut Arab University, and BLOM Bank. Hard copies of the questionnaire were also circulated amongst female employees and students at the Lebanese University, American University of Culture and Education, and Beit Baakline Primary Healthcare Center.

The study was approved by the Institutional Review Board of the American University of Beirut (AUB) and written consent forms were obtained from participants (registration number: SBS-2018-0450).

### Statistical analysis

Descriptive analysis was completed for all continuous and categorical variables, and a p-value of 0.05 or less was deemed an indicator of statistical significance. The primary outcome was a positive or negative attitude toward fertility preservation. To evaluate whether baseline characteristics of participants are associated with the attitudes toward fertility preservation, univariate and multivariate logistic regression analyses were completed to control the age group. All statistical analysis was completed using SPSS version 26.

## Results

### Demographics

A total of 402 women completed the survey and were included as participants in the study. The sociodemographic characteristics of respondents are outlined in Table 1. The participant’s ages ranged between 18 and 39 with 62% of them between 18 and 30. The majority were from a Muslim background, had advanced degrees, worked full-time with a monthly income of 1000–2000$, and were covered by governmental health insurance. The percentage of single women was 51.5% while the percentage of women that already had children was 66%.

**Table 1:** Sociodemographic characteristics of respondents.

	All women
Woman’s age	
18–24	36 (9)
25–34	276 (69)
35–39	88 (22)
Woman’s age	
18–30	248 (62)
31–39	152 (38)
Religious affiliation	
Christian	109 (27.3)
Muslim	248 (62)
Druze	24 (6)
Educational level	
High school or less	9 (2.3)
College Education	135 (33.8)
Advanced degrees	253 (63.3)
Working pattern	
Full time	382 (70.5)
Part-time	34 (8.5)
Student	62 (15.5)
Not employed	20 (5)
Monthly income	
Less than 1000$	98 (24.5)
1000–2000$	190 (47.5)
2000–5000$	69 (17.3)
More than 5000$	8 (2)
Insurance status	
No insurance	27 (6.8)
Governmental insurance	162 (51.8)
Private insurance	207 (40.5)
Relationship status	
Single	204 (51.5)
Married	176 (44)
Cohabiting	9 (2.5)
Divorced	10 (2.3)
Already has children	
Yes	264 (66)
No	135 (33.8)
Difficulty conceiving	
Yes	32 (8)
No	138 (34.5)
Not applicable	229 (57.3)

Data presented as n (%)

### Knowledge towards egg freezing

A total of 65% of the participants responded that they already heard about egg freezing, mainly from family/friends/common knowledge (26.1%). The results demonstrate that the majority of participants (67.7%) consider themselves as potential social oocyte freezers, of which 34.1% would consider the procedure (Table 2).

**Table 2:** Knowledge of egg freezing.

	All women
Have you heard about egg freezing?	
Yes	260 (65)
No	140 (35)
Where have you heard about egg freezing	
Family/Friends/common knowledge	104 (26.1)
Media	74 (18.6)
School/University/Scientific papers	50 (12.6)
Physician/IVF treatment	30 (7.5)
Did not hear of it	140 (35.2)
Would you consider freezing your eggs?	
No	69 (18)
Yes	131 (34.1)
Maybe	129 (33.6)
Do not know	52 (13.5)

Data presented as n (%)

### Attitudes toward medical and social egg freezing

The majority of respondents supported medical egg freezing with the highest percentage of acceptance (75.8%) being for fertility preservation before chemotherapy. The most supported indication for social egg freezing, on the other hand, was to allow a single woman who cannot guarantee that she will meet the right partner in time, to have children in the future (41.5%). All the other indications for social egg freezing mentioned in Table 3, had comparable percentages of acceptance.

**Table 3:** Circumstances that make egg freezing acceptable.

	Always acceptable	Sometimes acceptable	Not acceptable
Medical Reasons			
To allow women with chronic conditions to have children in the future	257 (64.3)	109 (27.3)	16 (4)
To increase the chance of women about to start treatment for cancer to have children in the future	303 (75.8)	76 (19)	4 (1)
To allow women who are having surgery on her ovaries, which may mean she would lose her ovaries, to have children in the future	293 (73.3)	80 (20)	9 (2.3)
To allow women with a known family history of very early menopause to have children in the future	260 (65)	113 (28.3)	10 (2.5)
Social reasons			
To allow a single woman who cannot guarantee that she will meet the right partner in time, to have children in the future	166 (41.5)	134 (33.5)	82 (20.5)
To allow a woman to postpone having children for career reasons	131 (32.5)	174 (43.5)	78 (19.5)
To allow a woman who cannot contemplate having a family now due to other care commitments, to have a family in the future	136 (34)	184 (46)	63 (15.8)
To allow a woman whose current relationship has failed and cannot guarantee that she will meet the right partner in time to have a family with, to have a family in the future	136 (34)	164 (41)	83 (20.8)
To allow a woman who is not ready to have children now, to have a family in the future	137 (34.3)	167 (41.8)	78 (19.5)

Data presented as n (%)

### Factors associated with considering egg freezing

After analysis of demographic factors associated with considering egg freezing, the sole statistically significant factor was whether the participant already had children or not, with the latter accepting egg freezing three times more aOR = 3.01 (95% CI = 1.44–6.29, p-value = 0.003, Table 4). Table 5 presents the different factors deemed important when considering potential egg freezing. Participants were divided into three groups based on attitude towards egg freezing: possible egg freezers, non-egg freezers, and those who remained indecisive. The most common unanimous concern for all three groups alike was whether the procedure would be proven safe for their future children, with potential egg freezers having the highest percentage (99.2%) when compared to non-freezers or those who remain uncertain respectively (90.2% and 98.3%, p-value = 0.003). The second most common concern was whether egg freezing would affect future fertility with the potential egg-freezing group and the indecisive group having more significant concerns (91.6% and 92.8% respectively) when compared to the non-egg freezers (78.7%, p-value = 0.005). As for the success rate of conception after freezing as a factor, a chance of over 50% of conception was deemed more important in the potential egg-freezers and indecisive groups (79.4% and 74% respectively) as opposed to the non-egg freezing group (57.4%, p-value = 0.006). The least important factor of all was the financial one, with 62% of potential egg freezers considering whether the procedure will be fully covered (rather than not) important when taking their decision. Around half of the indecisive group considered this financial aspect as not important compared to 44.3% of non-egg freezers (p-value = 0.03).

**Table 4:** Factors associated with considering egg freezing.

Women’s characteristics	(OR 95% CI)	p-value	aOR (95%CI)	p-value
Woman’s age				
18–30	2.09 (1.35–3.24)	0.001*	1.02 (0.59–1.72)	0.953
31–39	1			
Residency area				
Capital	0.98 (0.63–1.50)	0.915		
Outside Capital	1			
Religious affiliations				
Christian	1			
Muslim	1.39 (0.86–2.25)	0.176		
Druze	3.32 (0.64–4.87)	0.268		
Educational level				
College education or less	1			
Advanced degrees	1.45 (0.94–2.26)	0.096		
Working pattern				
Full-time	1.43 (0.82–2.52)	0.211		
Not full-time	1			
Income level				
Less than 1000$	1			
1000–2000$	1.35 (0.80–2.30)	0.266		
2000–5000$	1.06 (0.55–2.04)	0.865		
More than 5000$	0.54 (0.13–2.30)	0.406		
Relationship status				
Not in relationship	4.31 (2.71–6.86)	<0.001*	1.92 (0.95–3.88)	0.068
In relationship	1			
Has children				
No	5.00 (3.15–7.95)	<0.001*	3.01 (1.44–6.29)	0.003*
Yes	1			
Difficulty conceiving				
Difficulty conceiving	2.19 (0.92–5.22)	0.077		
No difficulty	1			

*Significant p-value<0.05

OR odds ratio, aOR adjusted odds ratio (only significate factors at a univariate level were included)

**Table 5:** Factors deemed important in considering egg freezing.

	All women	Egg freezers	Non-egg freezers	Indecisive	p-value
If you were to consider egg freezing, which of these would you need to be certain of before proceeding					
It will not affect my future fertility					
Important	336 (90.1)	120 (91.6)	48 (78.7)	168 (92.8)	0.005*
Not important	37 (9.9)	11 (8.4)	13 (21.3)	12 (7.2)	
It is proven safe for my future children					
Important	363 (97.3)	130 (99.2)	55 (90.2)	178 (98.3)	0.003*
Not important	10 (2.7)	1 (0.8)	6 (9.8)	3 (1.7)	
It will be fully paid for					
Important	172 (46.1)	49 (37.4)	34 (55.7)	89 (49.2)	0.03*
Not important	201 (53.9)	82 (62.6)	27 (44.3)	92 (50.8)	
There is more than a 50% chance I will get a baby					
Important	273 (73.2)	104 (79.4)	35 (57.4)	134 (74)	0.006*
Not important	100 (26.8)	27 (20.6)	26 (42.6)	47 (26)	
There is more than an 80% chance I will get a baby					
Important	290 (77.7)	99 (75.6)	46 (75.4)	145 (80.1)	0.567
Not important	83 (22.3)	32 (24.4)	15 (24.6)	36 (19.9)	
What personal circumstances will make it more likely for you to freeze your eggs					
If I have not found a partner yet by 35 years of age					
Important	195 (52.7)	86 (66.2)	19 (30.6)	90 (50.6)	<0.001*
Not important	175 (47.3)	44 (33.8)	43 (69.4)	88 (49.4)	
If I have not found a partner yet by 40 years of age					
Important	214 (57.4)	94 (71.8)	28 (44.4)	92 (51.4)	<0.001*
Not important	159 (42.6)	37 (28.2)	35 (55.6)	87 (48.6)	
If I have a partner, stopping work for children would harm my career					
Important	149 (39.9)	68 (51.9)	15 (23.8)	66 (36.9)	<0.001*
Not important	224 (60.1)	63 (48.1)	48 (76.2)	113 (63.1)	

Data presented as n (%)

*Significant p-value<0.05

When assessing the personal circumstances driving participants to consider egg freezing, 66.2% and 71.8% of potential egg freezers considered egg freezing if they have not found a partner yet by 35 and 40 years of age respectively. Both of these values were significantly higher when compared to both the indecisive and non-egg freezing groups, although they were also more likely to consider egg freezing after the age of 40 vs. 35 (44.4% vs. 30.6% and 51.4% vs. 50.6 respectively, p-value < 0.001).

Intentionally delaying childbearing (despite having a partner) due to perceived career threats, was a significantly more important driving force for freezing in the potential egg freezers group (51.9%), followed by the indecisive group (36.9%) and non-egg freezers (23.8%) (p-value < 0.001).

### Concerns regarding egg freezing

Table 6 lists the possible concerns hindering the participants from pursuing egg freezing. Interestingly, 62% of participants shied away from the procedure due to limited information on how the procedure is performed, making it the most prevalent concern. This was followed by the financial burden of egg freezing (48.5%), possible risks of hormonal treatment for ovarian stimulation (36%), and the ultimate possible complications of the procedure (26.8%). Conversely, cultural issues related to possible hymenal disruption during the procedure and embarrassment/ discomfort were the two least prevalent causes of apprehension regarding egg freezing (19% and 13% respectively).

**Table 6:** Concerns regarding egg freezing.

	n	percentage
Limited information on how the procedure is performed	248	62
Safety of procedure (bleeding, infection,..)	107	26.8
Possible risks of hormonal treatment for ovarian stimulation	144	36
Cultural issues related to possible disruption of the hymen during the procedure	76	19
Financial concerns regarding the cost of egg freezing	194	48.5
Possible Embarrassment/discomfort	52	13

## Discussion

Our study shed light on the attitudes of women in Lebanon towards social egg freezing. To our knowledge, this is the first endeavor to research attitudes toward social egg-freezing in the Lebanese population; thus, it plays an essential role in reducing the gap in knowledge surrounding this topic.

A total of 402 female participants, with ages ranging between 18 and 39, recruited from several organizations were integrated into the study. All three main religions in Lebanon were represented by the diverse recruited population. These religions have various reservations regarding egg freezing, ranging from the possibility of demeaning the divine process of reproduction to the religious dilemmas surrounding the whole IVF process [15]. Despite this traditional, religious outlook on the matter of ART, certain religious communities have begun to update their views on such dilemmas. For example, Dar al Ifta in Egypt, first deemed egg-freezing permissible for married Muslim women who were undergoing medical treatment that would threaten their ability to conceive (such as chemotherapy) [16], then later updated the fatwa (the Islamic law) to allow unmarried women to undergo egg-freezing under the condition that the eggs would not be damaged, and that they would only be fertilized within the confines of a marriage and during the husband’s lifetime [15]. These drastic changes in religious legislation have the potential to shift the overall Middle Eastern perspective towards acceptance of innovative treatments in ART.

The study also addressed women’s general acceptance and awareness regarding different concerns associated with oocyte cryopreservation. Our study shows that the percentage of participants that already heard about egg freezing (65%) is higher compared to populations studied in Italy (34.3%) [14], Dublin (60%) [17], and the US (29.8%) [13] but lower than that in the UK and Denmark (83%) [12]. These results portray that there is relatively a high percentage of awareness in the studied Lebanese population. One of the contributing factors to this high number is the high proportion of participants with advanced degrees (63%).

The results demonstrate that the majority of participants (68%) consider themselves as potential social oocyte freezers of which 34.1% would assuredly consider the procedure. These values were comparable to the German cohort (33%) [10] but were considerably higher when compared to other populations such as UK/Denmark (27%) [12], the US (21.6%) [13], and Italy (19.5%) [14]. Relatively younger women (18–30) were 2.09 times (95% CI: 1.35–3.24) more likely to consider egg freezing when compared to those with ages ranging from 31–39 years. In addition, participants that were not yet in a relationship were 4.31times (95% CI: 2.71–6.86) more likely to consider egg freezing than those who were in relationships, while childless women were 5.0 (95% CI: 3.15–7.95) times more likely to consider it than women with children (Table 4).

Overall, acceptance for medical egg freezing was higher when generally compared to social egg freezing. This is in line with previous research results in Australia [18], Sweden [19], and Canada [20] among others. The most accepted medical indication for egg freezing was before chemotherapy (75.8%) followed by pre-ovarian surgery (73.3%). The acceptance of medical egg-freezing in patients with chronic medical conditions or a history of very early menopause were congruous (64.3% and 65% respectively, Table 3). As for social egg freezing, the most supported indication was to allow single women who cannot guarantee meeting a suitable partner in time to have children in the future (41.5%). All the other indications for social egg freezing mentioned in Table 3 had comparable percentages of acceptance (34% for women who couldn’t contemplate having children due to other care commitments and women who had previous failed relationships and worried about finding a suitable partner in time to have children, 34.3% for women who weren’t currently ready to have children, and 32.5% for women who wanted to postpone having children for career reasons). This prevailing entity of higher acceptance of social egg freezing because of a lack of a suitable partner compared to career advancement is consistent across different population cohorts such as Australia (75% vs. 65%) [18] and UK/Denmark(85% vs 66%) [12]. The only study with discrepant results was that assessing a US cohort which showed a higher acceptance of social egg-freezing for career advancement (72.1) vs. lack of partner (63%) [21]. This lower acceptance of career advancement as an indication for social egg-freezing could stem from the negative and judgmental portrayal of career-pursuing females as “self-absorbed” individuals intentionally avoiding motherhood as elaborated by Mertes et. al [22]. This opinion could emanate from the deeply rooted traditional nature of the Lebanese population despite it being the most liberal in the Middle East.

When assessing factors affecting the decision to pursue egg freezing, the most common unanimous concern for all three groups alike (displayed in Table 5) was whether the procedure will be proven safe for their future children, with potential egg freezers having the highest percentage (99.2%) when compared to non-freezers or those who remain uncertain respectively (90.2% and 98.3%, p-value = 0.003). Recently, a growing body of research has been conducted on the neonatal outcomes and long-term follow-up after frozen-thawed embryo transfer. Gullo et al. outlined insights into neonatal outcomes, such as birth weight, gestational age, and congenital anomalies. A comparison was made between children born following frozen embryo transfers and those born following fresh embryo transfers. Potential differences in obstetric and neonatal outcomes between these groups were explored [23]. In addition, Gullo et al. suggest no direct evidence is found on the long-term follow-up of children conceived through frozen-thawed embryo transfer, assessing their developmental and health outcomes compared to naturally conceived children. The findings in the mentioned studies appear reassuring to couples aiming for cryopreservation techniques [24].

The second most common concern was whether egg freezing will affect future fertility with the potential egg-freezing group and the indecisive group having more significant concerns (91.6% and 92.8% respectively) when compared to the non-egg freezers (78.7%, p-value = 0.005). The least important factor of all was, surprisingly, the financial cost, 62% of potential egg freezers considered whether the procedure will be fully covered or not as important when making their decision. Around half of the indecisive group considered this financial aspect as not important compared to 44.3% of non-egg freezers (p-value = 0.03). The limited importance of the cost of egg freezing could be attributed to the relatively higher socioeconomic status of the participants involved and to the significantly lower cost of any ART treatment in Lebanon as compared to other countries (the maximum cost of 4000–5000$).

When tackling concerns hindering acceptance of the procedure in a rather relatively conservative country, one would expect the most prevalent concerns driving patients to shy away from the procedure would be cultural issues related to possible hymenal disruption during the procedure. As it turned out, this reason was found to be among the least prevalent (19%). The most common concern was having limited information on how the procedure is performed (62%). This concern highlights the importance of proper awareness and education on the matter which is the responsibility of gynecologists during routine visits. The second most common was the financial burden of egg freezing (48.5%), followed by possible risks of hormonal treatment for ovarian stimulation (36%), and the ultimate possible complications of the procedure (26.8%).

## Limitations

While this study played an important role in expanding the research on women’s attitudes towards egg freezing for non-medical reasons, it is not without its limitations. The majority of the women included in this study held a college degree, with 63.3% of them holding advanced degrees. Concurrently, 51.5% of the subjects were single. It is logical then, to assume potential bias in the women’s attitudes that favors a positive outlook towards social egg-freezing due to their career-oriented backgrounds, single marital status, and the potential opportunities that social egg-freezing would provide them.

Furthermore, by excluding men and restricting the study’s subjects to one gender, we were only able to analyze the opinions of the female half of Lebanon’s population. The attitude of Lebanese men towards social egg-freezing remains unknown; by default, the implications, and effects of their attitudes on the overall societal view towards social egg freezing is also unclear.

Finally, follow up studies are necessary to explore whether positive attitudes towards social egg freezing translate in practice to an increase in the number of women undergoing this procedure.

## Conclusion

The present study addresses a significant gap in literature by examining the awareness and acceptance of social egg freezing in the Lebanese population. However, our findings highlight a gradual shift in public opinions towards emphasizing the conscious reproductive autonomy in a relatively conservative country. Nonetheless, limited information about the procedure emerges as a significant obstacle to achieving even higher acceptance rates. The findings suggest that physicians and primary healthcare providers can play a crucial role in educating women of reproductive age and providing them with up-to-date information about the available modalities that can safeguard their reproductive potential.
